# Impact of COVID-19 pandemic on road traffic injuries in Iran: An interrupted time-series analysis

**DOI:** 10.1371/journal.pone.0305081

**Published:** 2024-06-17

**Authors:** Pirhossein Kolivand, Peyman Saberian, Jalal Arabloo, Masoud Behzadifar, Fereshteh Karimi, Soheila Rajaie, Morteza Moradipour, Arash Parvari, Samad Azari

**Affiliations:** 1 Faculty of Medicine, Department of Health Economics, Shahed University, Tehran, Iran; 2 Department of Anesthesiology, Imam Khomeini Hospital Complex, Tehran University of Medical Sciences, Tehran, Iran; 3 Health Management and Economics Research Center, Health Management Research Institute, Iran University of Medical Sciences, Tehran, Iran; 4 Social Determinants of Health Research Center, Lorestan University of Medical Sciences, Khorramabad, Iran; 5 Research Center for Emergency and Disaster Resilience, Red Crescent Society of the Islamic Republic of Iran, Tehran, Iran; 6 Department of Epidemiology and Biostatistics, School of Public Health, Tehran University of Medical Sciences, Tehran, Iran; 7 Hospital Management Research Center, Health Management Research Institute, Iran University of Medical Sciences, Tehran, Iran; Cairo University, EGYPT

## Abstract

**Introduction:**

Globally, the COVID-19 pandemic has affected the number of road accidents and deaths caused by them. The present study aimed to identify the effect of this epidemic on traffic accidents and their casualties in Iran.

**Methods:**

In this study, Interrupted Time Series Analysis (ITSA) was used in a semi-experimental design to measure the impact of the restrictive policies of COVID-19 on road accidents. Data were collected retrospectively from the Iran Red Crescent Society data set for 31 provinces from March 2017 to February 2022. The information related to the number of road accidents, injuries, deaths, and deaths in the hospital was collected. The Newey‐West method is used for estimation. Statistical analyses were carried out using R software version 3.6.1.

**Results:**

Since February 2020 in Iran, the reduction in the number of road accidents and the number of injuries and deaths in these accidents was significant at 5% but the reduction of deaths in the scene and hospital was significant at 10%. In general, for all variables, the reduction trend was established only in the first months, and then it had an upward trend.

**Conclusion:**

In the early months of the COVID-19 epidemic in Iran, the number of road accidents and their casualties decreased. Policies restricting traffic, quarantine, and fines for violators can be reasons for changing people’s behavior and travel patterns and also lead to a reduction in traffic accidents and fatalities. Such studies can explain the importance of the policies in changing behavioural patterns and can be used as a guide in future policies.

## 1. Introduction

The COVID-19 pandemic has impacted travel patterns, road congestion, and road travel safety. Road accidents are still considered as one of the most significant traffic problems in the world, which cause financial losses, economic challenges, injuries and fatalities. In response to the rapid spread of the COVID-19 pandemic, regional and global movement restrictions have brought about fundamental changes in people’s travel behavior [1].

There might be several reasons for this event. By the start of the quarantine, movements decreased and the worry about being exposed to the virus also decreased. With reduced movements, the number of cars on the streets decreased and the risk of accidents also decreased as a result. Therefore, traffic has decreased and drivers have found the opportunity to increase their speed in the quiet streets. Speeding is a very important factor in accident deaths [2].

COVID-19 also has led to an increase in unemployment. Empirical evidence suggests that movement and accident rates decrease during recessions [3]. Research has shown that financial worries and economic uncertainty increase distraction, frustration, and sleep deprivation, have positive relation with accidents and potentially play role in roads accidents. Moreover, inequality and financial concerns may decrease social cohesion and be considered as a reason for selfish behaviurs [4]. Being concerned about the infection of relatives due to COVID-19 can also lead to distraction and raise the risk of road accidents [5]. Furthermore, disturbances in sleep patterns and changes in alcohol consumption have affected the rate of road accidents during the COVID-19 pandemic [6,7].

The results of the global studies on the impact of the COVID-19 pandemic on road accidents globally are divergent. In the United States, several studies have indicated a decrease in the overall number of accidents and people involved in road accidents. However, there has been an increase in Angle crashes, while the severity and fatality rates remain unchanged [8–12]. In Greece, while the total number of traffic accidents decreased, there was a significant increase in both fatalities and minor injuries [13]. In China, while the total number of road traffic incidents decreased, there was a rise in road traffic injuries and fatalities specifically involving e-bikes [14,15].

Studies have investigated the impact of the COVID-19 pandemic on road accidents using various methods and perspectives. For example, in Japan, the impact of speeding violations during quarantine was predicted by analyzing police data on the monthly number of fatal motor vehicle accidents (MVC) from January 2010 to February 2020 in which drivers were at fault. The study revealed that in February 2020, there were more speeding violations during quarantine compared to the initially predicted rate [16].

A study combined detailed information like motor vehicle travel, fatalities, crash, fatality, and weather data, and driver characteristics to analyze the impact of the COVID-19 pandemic on road safety in California. Data were analyzed from March 1, 2015, to May 31, 2020. The study calculated the mean values of driver and crash characteristics before and during the initial period of COVID-19 to interpret differences as evidence of compositional changes during driving restrictions. The results of the study showed that during the first eleven weeks of the reaction to COVID-19, driving in the highways decreased by approximately 22% and total crashes decreased by 49%. Although the statewide average speed increased by 2 to 3 mph, certain counties experienced a more significant increase of 10 to 15 mph. The proportion of severe crashes increased by approximately 5%, representing a 25% rise [17].

The study by Zhang et al. investigated the impact of COVID-19 on the number of people involved in accidents in New York from January 1, 2020, to May 24, 2020, using the negative binomial method. The study finds that different levels of control policies during the COVID-19 outbreak are closely related to safety awareness, and driving and travel behaviour, and indirectly affect the frequency of accidents. In particular, a negative correlation was identified between the implementation of the stay-at-home policy in New York City on 20 March 2020 and the number of individuals involved in the accidents [12].

Iran is a middle-income country, and its road accidents and their burden have always been a great concern. According to the latest report of The Global Burden of Disease (GBD) study in 2019, there were 21,121 deaths due to road accidents. During the COVID-19 epidemic in 2020, there was a 29% decrease in intercity mobility in Iran [18]. A study revealed that in 2020 and 2021, an average of 15,000 people died in road accidents annually [19]. Furthermore, a study in Shiraz compared the prevalence and causes of death among patients admitted due to road accidents during the COVID-19 epidemic with the period before. The results indicated a decrease in the number of patients compared to before, but an increase in the number of deaths from road accidents [20].

Although studies in Iran have investigated various aspects of this issue, but none of them have examined all the country’s provinces simultaneously. This study was carried out to investigate the change in the pattern of road accidents in Iran during the COVID-19 epidemic. This study also investigated changes in the number of road accidents during the COVID-19 pandemic compared to the period before and after it in Iran for the first time. In addition to the number of road accidents, the number of deaths at the scene of the accident and medical centers, and therefore the change in the severity of fatalities of accidents, has also been investigated in the periods before and after this epidemic. The results of this study can show the effect of applied policies on changing people’s behavior, patterns of accidents and fatalities in Iran during the Covid-19 pandemic. The results and lessons learned from these policies related to this disease can be considered as a useful guide in planning and future transportation policies.

## 2. Material and methods

### 2.1 Ethics statements

This study is an extract from the research project with the Code of Ethics IR.RCS.REC.1401.014 of the Iranian Red Crescent Society, which has been conducted at the Research Centre for Emergency and Disaster Resilience, Red Crescent Society of the Islamic Republic of Iran, Tehran, Iran.

### 2.2 Study design

This study utilized interrupted time series analysis (ITSA) in a quasi-experimental design. The study addresses the interest of policymakers in assessing the effects of various health sector policies. Depending on the outcomes observed, policymakers can decide whether to maintain or discontinue these policies [21]. ITSA is a widely used method for assessing the effectiveness of clinical and non-clinical interventions and providing valuable evidence for decision-making [22]. ITSA involves examining a continuous series of observations over time to understand the effects of implementing a policy or intervention [23]. By comparing the before-and-after periods, ITSA contributes to a deeper understanding of the policy’s effects [24]. In the context of health crises such as COVID-19, ITSA plays a crucial role in evaluating the consequences of different policies and helping managers in make well-informed decisions [25].

### 2.3 Data collection

The information of this study was collected from the database of the Iranian Red Crescent Relief and Rescue Organization. This organization has aid stations in the whole country and records the information on accidents on all the country’s roads. This information is recorded in a special form and records the exact details such as the location and time of the accident, the type of vehicles, the number of casualties and injuries at the accident scene, the number of services and the type of assistance at the accident scene, and the number of survivors. The authors did not have access to information that could identify individual participants during or after data collection.

After receiving approval from the Iranian Red Crescent Research Ethics Committee under the number IR.RCS.REC.1401.014, the required data were obtained in the specified time frame from March 2017 to February 2022 and subsequently organized by the authors in a structured format based on research objectives. The appearance of COVID-19 in Iran was in February 2020. The study investigated the impact of the COVID-19 epidemic in Iran on various indicators, including the number of road accidents, injuries, and deaths both at the scene and in hospitals.

### 2.4 Statistical analysis

The ITSA will involve fitting a segmented regression model to the data. The model specification is as follows:

$$Y_{t}=\beta_{0}+\beta_{1}*Time+\beta_{2}*Intervention+\beta_{3}*Time*Intervention+\varepsilon_{t}$$

Y_t:_ represents the number of indexes at time t

Time: is a continuous variable representing the time in years.

Intervention: is a binary variable indicating the presence [26] or absence (0) of the COVID-19 pandemic interventions

β_0:_ represents the intercept, representing the level of index at the beginning of the study period

β_1:_ estimates the baseline trend in the index before the intervention

β_2:_ captures the index immediate change in the level after the intervention

β_3:_ estimates the difference in the trend after the intervention compared to the pre-intervention period

ε_t:_ represents the error term assumed to be normally distributed

We employed a segmented regression model in our analysis of ITSA. To ensure the best analytical approach, we employed the Newey‐West method for estimation. We conducted various diagnostic and sensitivity assessments to verify the strength of our findings. To identify any serial correlation in errors, we employed the ordinary least squares (OLS) regression model with a time series specification and the Durbin-Watson test. We also assessed residuals from the OLS regression and examined autocorrelation and partial autocorrelation plots. To ensure that the assumptions of our regression model are met, we verified that the error term is normally distributed and represents independent and identically distributed (IID) characteristics. Diagnostic tests, including the ordinary least squares (OLS) regression model with a time series specification, the Durbin-Watson test to identify serial correlation in errors, and examination of autocorrelation and partial autocorrelation plots of residuals, were conducted to assess the Gaussian white noise characteristics of the residuals. We employed the Box-Jenkins approach, which considers seasonal correlations, such as regression to mean effects. Box—Jenkins introduced the ARIMA model, which integrates both autoregressive (AR) and moving average (MA) models. Additionally, the model explicitly incorporates differencing in its formulation. The AR component characterizes a time series where the present observation relies on its past values, while the MA component depicts a time series as a linear function of current and past random errors. All statistical analyses were carried out using R software version 3.6.1 via *nlme* package. A significance level of P-value < 0.05 was adopted.

## 3. Results

The results of the study were presented separately in 4 sections: The number of road accidents, injuries, road accidents deaths at the scene of the accident and the total number of road accident deaths at the scene and hospital.

### 3.1. The number of road accidents

During the first month of the COVID-19 pandemic in Iran, a significant decrease in the number of road accidents was observed, amounting to 519 cases (95% CI: 239.29–800.45, P < 0.001). The results of the regression model are shown in Table 1.

**Table 1:** The segmented regression model for the number of road accidents.

Regression with Newey West Standard Errors Maximum Lag: 1 *F*_3,56_ = 24.91 Probability > *F* = 0.00 Number of Observations = 60					
Parameter	Coefficients	Newey‐West Standard Errors	*P* Value	Lower of CI (95%)	Upper of CI (95%)
Intercept	2011.48	91.47	0.00	1828.22	2194.73
Pre-intervention slope	-6.26	5.35	0.24	-16.98	4.46
Change in intercept	-519.87	140.06	0.00	-800.45	-239.29
Change in slope	5.34	7.98	0.50	-10.66	21.34
Post-intervention linear trend	-0.92	5.28	0.86	-11.50	9.66

**Fig 1:**
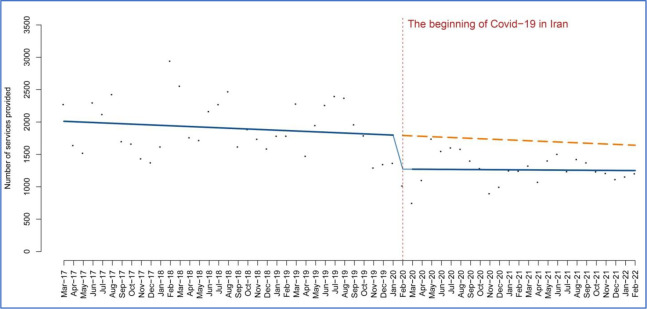
The temporal trend of the rate of road accidents rate in Iran.

The ITSA revealed an immediate reduction of 0.92 in road accidents (95% CI 9.66 to 11.50; P = 0.86) following the onset of COVID-19. However, the decreasing trend in road accidents did not persist after the initial decline in the first months. Instead, the number of road accidents started to increase again, indicating a continuing upward trend (5.34; 95% CI 10.66 to 21.34; P = 0.50). The OLS regression of the rate of road accidents in Iran is depicted in Fig 1. This means that the downward trend in traffic accidents has continued, while the slope has not been as steep as in the pre-intervention period.

### 3.2. The number of injuries in road accidents

In the month following the announcement of COVID-19 in Iran, a significant decrease in the number of injuries caused by road accidents was observed. There was a reported reduction of 1602.67 cases (95% CI: 899.39–2305.95, P < 0.001). The results from the regression model are available in Table 2.

**Table 2:** Temporal trend of people injured in road accidents.

Regression with NeweyWest Standard Errors Maximum Lag: 1 *F*_3,56_ = 75.22 Probability > *F* = 0.00 Number of Observations = 60					
Parameter	Coefficients	Newey‐West Standard Errors	*P-Value*	Lower of CI (95%)	Upper of CI (95%)
Intercept	4599.25	220.14	0.00	4158.25	5040.24
Pre-intervention slope	-11.25	13.39	0.40	-38.09	15.58
Change in intercept	-1602.67	351.07	0.00	-2305.95	-899.39
Change in slope	18.73	18.75	0.32	-18.84	56.30
Post-intervention linear trend	7.47	12.38	0.54	-17.33	32.29

**Fig 2:**
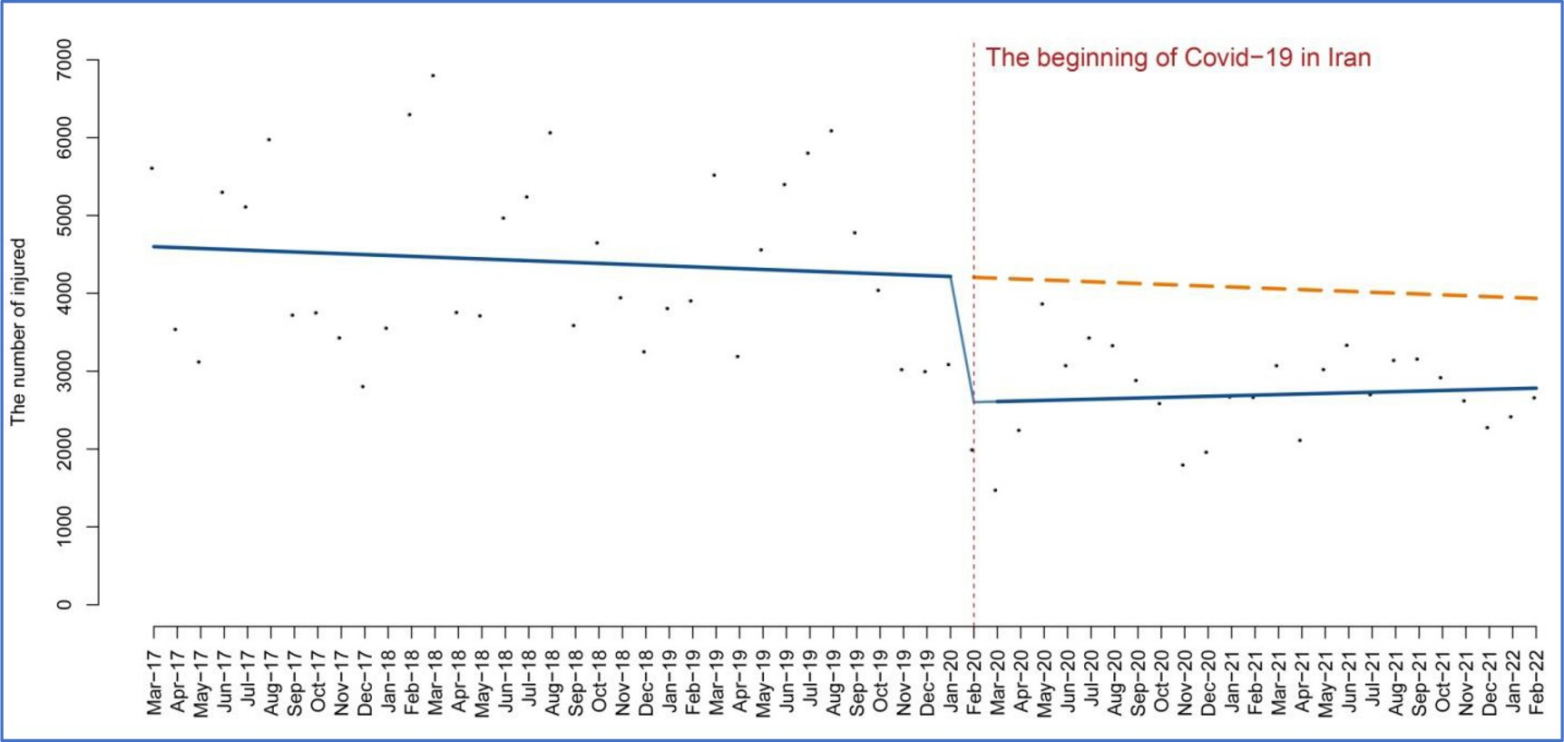
The temporal trend of accidents caused by roads in Iran.

However, despite the initial decrease, a subsequent upward trend in the number of injuries resulting from road accidents was observed over time. The increase was measured at an incremental rate of 7.47 (95% CI 17.33 to 32.29; P = 0.54). Fig 2 shows the temporal trend of the number of injuries caused by road accidents in Iran before and after COVID-19. This means that the downward trend in traffic accidents has continued, while the slope has not been as steep as in the pre-intervention period.

### 3.3. The number of deaths caused by road accidents at the scene of the accident

After the onset of COVID-19 in Iran, a decrease in the number of deaths at the scene of road accidents was observed, with a rate of 85.51 per 100,000 people (95% CI 50.35 to 120.67; P = 0.00). The results from the regression model are available in Table 3.

**Table 3:** Results from the segmented regression model for the number of deaths caused by road accidents at the scene of the accident.

Regression with NeweyWest Standard Errors Maximum Lag: 1 *F*_3,56_ = 47.74 Probability > *F* = 0.00 Number of Observations = 60					
Parameter	Coefficients	Newey‐West Standard Errors	*P* Value	Lower of CI (95%)	Upper of CI (95%)
Intercept	258.25	9.01	0.00	240.20	276.31
Pre-intervention slope	0.16	0.57	0.77	-0.98	1.30
Change in intercept	-85.51	17.55	0.00	-120.67	-50.35
Change in slope	1.03	0.85	0.23	-0.67	2.75
Post-intervention linear trend	1.20	0.69	0.08	-0.19	2.59

**Fig 3:**
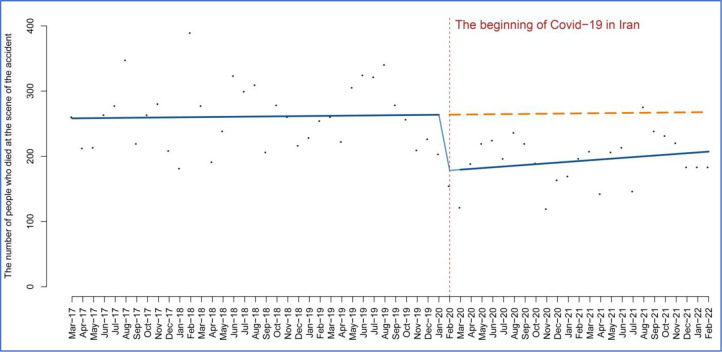
The temporal trend of the number of deaths at the scene of road accidents in Iran.

Despite the initial downward trend, deaths started to increase again, and this increase was found to be statistically significant, measuring at 1.20 (95% CI 0.19 to 2.59; P = 0.08). Fig 3 illustrates the temporal trend of the number of deaths at the scene of road accidents in Iran before and after COVID-19. This indicates that the downward trend in traffic accidents has continued, although the slope has not been as steep as in the preintervention period.

### 3.4. The total number of deaths from road accidents at the scene and the hospitals

In the first month after the start of COVID-19 in Iran, there was a decrease in the rate of deaths at the scene of road accidents and after being admitted to hospitals, with a rate of 2.42 per 100,000 people (95% CI 0.42 to 5.28; P = 0.09) (Table 4).

**Table 4:** Results of the segmented regression model for the number of road accident deaths at the scene and hospitals.

Regression with NeweyWest Standard Errors Maximum Lag: 1 *F*3,56 = 7.12 Probability > *F* = 0.00 Number of Observations = 60					
Parameter	Coefficients	Newey‐West Standard Errors	*P-* value	Lower of CI (95%)	Upper of CI (95%)
Intercept	260.65	8.39	0.00	243.83	277.46
Pre-intervention slope	0.08	0.52	0.87	-0.97	1.14
Change in intercept	-10.51	33.87	0.75	-78.37	57.34
Change in slope	-2.51	1.37	0.07	-5.27	0.23
Post-intervention linear trend	-2.42	1.42	0.09	-5.28	0.42

This downward trend in deaths persisted in the subsequent months, with a decrease of 10.51 (95% CI, 78.38 to 57.34; P = 0.75).

**Fig 4:**
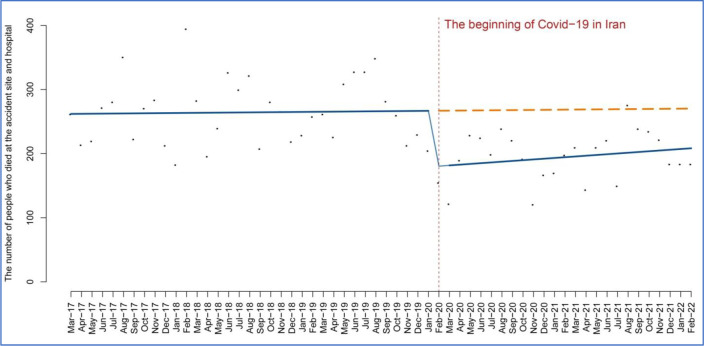
The temporal trend rate of deaths at the scene of road accidents and after admission to hospitals in Iran.

Fig 4 shows the death temporal trend of the rate of deaths at the scene of road accidents and after being admitted to hospitals in Iran before and after COVID-19. This means that the downward trend in traffic accidents has continued, while the slope has not been as steep as in the pre-intervention period.

The findings obtained through the Box-Jenkins approach are presented in Table 5.

**Table 5:** Results from the Box-Jenkins approach.

	Number of road accidents	Injured in road accidents	Number of deaths caused by road accidents at the scene of the accident	Number of deaths from road accidents at the scene and hospitals
ar1	0.4642	-0.3737	-0.5344	
ma1	-0.9045	-0.3647	-0.2530	-0.6751
sar1	0.4734	0.5193	0.4312	0.4658
Log likelihood	-422.97	-481.16	-309.2	-311.04
AIC	853.93	970.32	626.4	628.08
AICc	854.67	971.06	627.14	628.52
BIC	862.24	978.63	634.71	634.31
ME	-45.41607	-86.14291	-1.934584	-1.160256
RMSE	302.2007	804.9153	44.08247	45.35741
MAE	224.2191	633.1652	34.03095	35.07435
MPE	-5.82074	-6.414571	-4.135401	-3.801491
MAPE	15.14516	19.46461	15.79844	16.31387
MASE	0.7063874	0.72171	0.7205495	0.7429694
ACF1	0.00644152	0.02212911	-0.02724557	0.08936564

(ar1: Autoregressive coefficient 1, ma1: Moving average coefficient 1, sar1: Seasonal autoregressive coefficient 1, AIC: Akaike Information Criterion, AICc: Correction to Akaike Information Criterion, BIC: Bayesian Information Criterion, ME: Mean error, RMSE: Root Mean Squared Error, MAE: Mean Absolute Error, MPE: Mean Percentage Error, MASE: Mean Absolute Scaled Error, ACF1: First autocorrelation coefficient)

The results shown in Table 5 suggest that the spread of Covid-19 has contributed to a reduction in the indicators mentioned. However, it is important to note that these indices alone may not provide conclusive evidence of the causal effect of Covid-19.

## 4. Discussion

This study was conducted to investigate the impact of COVID-19 on road accidents in Iran. According to the results in the initial months and after the announcement of COVID-19, quarantines and movement restrictions, there was a decrease in the number of accidents and deaths in Iran. Iran implemented policies for international and intercity travel. On 6 March 2020, the social distancing policy of 1.8 meters was implemented in all towns. All public places, schools, universities, cinemas and national sports halls were closed. The hours of work were also reduced [27]. Additionally, commercial activities were divided into four groups, and a smart and dynamic policy was adopted so that certain groups are not allowed to operate at specified intensity levels. The analysis showed the effectiveness of this policy, as it significantly reduced mortality due to Covid during the first peak [28].

On 21 November 2020, the government implemented a new intervention called the Smart Travel Ban (STB) policy. According to this policy, only the drivers who intended to travel between towns for commercial and essential purposes were allowed to travel. Drivers of private cars should take the traffic ban seriously and face significant fines if they drive on intercity highways. The results show that the (STB) policies reduced intercity travel by 29% [18].

Hence, at the onset of the COVID-19 epidemic, policies restricting movement within and between cities, closure of public places and many businesses, coupled with public apprehension regarding unnecessary travel and disease transmission, have influenced the patterns of road accidents and deaths in Iran. The study results indicated that over time, these patterns evolved as restrictions eased. Stringent and restrictive traffic policies have proven successful in reducing traffic accidents and casualties, consequently.

COVID-19 affected road accidents and the severity of fatalities in various ways, with effects varying according to the stringency of restrictions and policies across countries. The reduction in the number of vehicles on the roads due to restrictions and quarantines influenced road safety and accident rates significantly. Many studies have reported a decrease in the number of road accidents during quarantine periods. However, the number of fatalities and the severity of incidents exhibited variations [1].

According to data from The International Traffic Safety Data and Analysis Group (IRTAD), in OECD countries, many governments implemented stringent restrictions on nonessential movements in early 2020. Initially, most countries had high stringency levels that later fluctuated. These restrictions significantly influenced the number and patterns of accidents. In most countries, traffic volumes have decreased since March 2020, with the most significant declines observed in April and May 2020. According to the report, traffic volume in 2020 was 12.2% lower than the average volume recorded from 2017 to 2019. In general, the number of road deaths in 2020 across the 34 IRTAD countries decreased by 8.6%. [29]. The results of these reports are also relevant to the present study and confirm the results of our study.

In the United States, COVID-19 has influenced people’s behavior and road accidents. During this period, there has been a decrease in both the number of traffic accidents and the individuals involved in such incidents. However, despite these changes, some individuals have reported maintaining their pre-pandemic behavior. Consequently, it appears that the imposed restrictions may have led to an increase in risky driving behaviour among some individuals. This manifested in drivers exhibiting more reckless and faster driving habits, potentially contributing to the persistence of serious and fatal road accidents [8–12,26].

In Canada, during traffic lockdown, there has been a decrease in deaths and injuries among older drivers, but there has been an increase in risky behaviors [26,30]. One of the reasons is that the elderly followed social distancing rules more because they were more concerned about being exposed to the disease. This led to a reduction in casualties and deaths from traffic accidents in this age group [2].

In Spain, due to the reluctance to use public transport, more private vehicles were driven and the number of road accidents increased [31,32]. In China, there was an increase in traffic injuries caused by e-bikes. One possible reason for the rise in personal vehicle accidents could be attributed to the COVID-19 era. Social distancing rules and concerns about virus exposure in public spaces led to a surge in the use of personal transportation modes like private cars, cycling, and walking, while the utilization of public transportation such as taxis and buses significantly decreased [1].

During COVID-19, shopping was one of the main reasons for leaving the house. Quarantine measures prompted a shift towards online activities, including purchasing essential supplies and e-books online to reduce the need for physical movement. The pandemic has compelled communities to reassess road infrastructure and create safe environments for walking and cycling [1].

This study had some limitations. First, all data were sourced from the Iranian Red Crescent Relief and Rescue Organization, which means information from other emergency organizations is not included. Additionally, caution should be exercised when attributing all reductions in accident numbers solely to restrictive policies. Many people voluntarily curtailed their travels out of concern for the health of their own and their loved ones.

## 5. Conclusion

During the COVID-19 epidemic, the number of traffic accidents and deaths in Iran has decreased. By being aware of its results and analyzing the country’s policies and people’s behavior, policymakers can apply the lessons learned from this epidemic in future policies to reduce the traffic accidents.

## Supporting information

S1 File (XLSX)
